# Differences in Hyperandrogenism Related to Early Detection of Non-Classical Congenital Adrenal Hyperplasia on Second Newborn Screen

**DOI:** 10.3390/ijns9030050

**Published:** 2023-09-01

**Authors:** Bonnie McCann-Crosby, Mark C. Liang, Mitchell E. Geffner, Christina M. Koppin, Nicole R. Fraga, V. Reid Sutton, Lefkothea P. Karaviti, Gagandeep Bhullar, Mimi S. Kim

**Affiliations:** 1 Texas Children’s Hospital, Baylor College of Medicine, Houston, TX 77030, USA; 2 Children’s Hospital Los Angeles (CHLA), Los Angeles, CA 90027, USA; 3 The Saban Research Institute at CHLA, Los Angeles, CA 90027, USA; 4 Keck School of Medicine, University of Southern California, Los Angeles, CA 90033, USA; 5 Department of Molecular and Human Genetics, Texas Children’s Hospital, Baylor College of Medicine, Houston, TX 77030, USA

**Keywords:** non-classical congenital adrenal hyperplasia, 21-hydroxylase deficiency, second newborn screen, genotyping

## Abstract

Screening for congenital adrenal hyperplasia (CAH) remains heterogenous across geographies—we sought to determine the proportion of non-classical CAH (NCAH) detection by one vs. two newborn screens (NBS) in two U.S. regions. Data were collected at tertiary centers in Houston (HOU) and Los Angeles (LA) on 35 patients with NCAH, comparing patients identified via the NBS vs. during childhood, 17-hydroxyprogesterone (17-OHP) levels, genotype, and phenotype. The NBS filter-paper 17-OHP levels and daily cutoffs were recorded on initial and second screens. In all, 53% of patients with NCAH in the HOU cohort were identified as infants via the second NBS. Patients identified clinically later in childhood presented at a similar age (HOU: *n* = 9, 5.5 ± 3.1 years; LA: *n* = 18, 7.9 ± 4 years) with premature pubarche in almost all. Patients in LA had more virilized phenotypes involving clitoromegaly and precocious puberty and were older at treatment onset compared with those identified in HOU by the second NBS (HOU: 3.2 ± 3.9 years; LA: 7.9 ± 4.0 years, *p* = 0.02). We conclude that the early detection of NCAH could prevent hyperandrogenism and its adverse consequences, with half of the cases in HOU detected via a second NBS. Further studies of genotyping and costs are merited.

## 1. Introduction

Congenital adrenal hyperplasia (CAH) is a group of autosomal recessive disorders characterized by defective cortisol biosynthesis. Over 90% of cases are caused by variants in the CYP21A2 gene, resulting in a deficiency of 21-hydroxylase and the subsequent impairment of cortisol production, accumulation of steroid precursors, and increased production of androgens [1]. The age at which clinical manifestations first appear varies widely depending on the degree of impaired enzyme activity. CAH is clinically classified based on the severity of symptoms noted at initial presentation [2], including two classical, severe forms of CAH (salt wasting (SW) and simple virilizing (SV)) and the mild, late-onset form, non-classical CAH (NCAH). NCAH has an overall prevalence of 1 in 1000, affecting 1 in 200 individuals in various Caucasian populations, and includes a spectrum of clinical symptomatology [3,4]. Clinical manifestations of NCAH in children can include signs of excess androgen production: premature pubarche, advanced bone age, and rapid height gain [5]. In contrast to patients with classical CAH, patients with NCAH may not necessarily require immediate treatment, although glucocorticoid replacement therapy could minimize the adverse effects of hyperandrogenism during childhood [5,6]. However, insulin resistance is commonly seen in adults with NCAH, along with hirsutism in women with NCAH [7]. Short stature can be a part of the clinical phenotype in adults, with 7% of pediatric NCAH patients exhibiting a predicted adult height SDS of ≤−2.0 [6,7,8]. The effects on final adult height vary in reports, from no differences in final adult height in treated and untreated NCAH patients in Italy to a mean height standard deviation score (SDS) of −0.4 ± 0.9 in adults with NCAH in the U.S. [7,9].

Newborn screening (NBS) for CAH secondary to 21-hydroxylase deficiency is based on the detection of elevated 17-hydroxyprogesterone (17-OHP) levels from eluates of dried blood spots on filter paper. In 1977, Alaska was the first state in the United States (U.S.) to perform NBSs for CAH, with the goal of identifying cases of classical CAH due to 21-hydroxylase deficiency [10]; Texas initiated NBSs for CAH in 1989, and California followed later in 2005. As of 2008, all states and territories in the U.S. perform mandated NBSs for CAH due to 21-hydroxylase deficiency [11]. In addition, there are 14 states performing a second NBS between 7 and 14 days of life, including Texas. NBSs for CAH are available in dozens of countries worldwide; however, they are neither universal nor mandatory in most.

Although the NBS is designed to identify cases of classical CAH due to 21-hydroxylase deficiency, previous studies have shown that NCAH can be detected by 17-OHP screening after birth, with a majority of cases identified on a second screen 1–2 weeks after the first [12,13,14]. While NCAH can be identified in the newborn period, little is known about how the 17-OHP levels, genotype, and clinical outcomes due to hyperandrogenism may differ between patients identified early in life (via the NBS) and those diagnosed later in childhood. The early detection of patients with NCAH could lead to closer monitoring for signs of excess androgen production and identify those patients who would benefit from early hormone replacement therapy.

Therefore, we aimed to identify the percentage of patients with a diagnosis of NCAH due to 21-hydroxylase deficiency made on one vs. two newborn screens in Texas and California pediatric tertiary centers. We also sought to compare NBS 17-OHP levels and clinical outcomes in patients with NCAH diagnosed via the NBS compared to patients diagnosed clinically later in childhood.

## 2. Materials and Methods

The protocol for this study was approved by the Institutional Review Boards at Baylor College of Medicine—Texas Children’s Hospital (TCH, Houston, TX, USA), the Texas Department of State Health Services, Children’s Hospital Los Angeles (CHLA, Los Angeles, CA, USA), and the Genetic Disease Screening Program at the California Department of Public Health. Informed consent was obtained for all patients prior to reviewing their NBS results.

This was a retrospective, cross-sectional study at the two tertiary pediatric centers; TCH and CHLA both serve as referral sites for newborn screening in their respective states. Each center identified patients with a diagnosis of NCAH due to 21-hydroxylase deficiency who had been seen between 1995 and 2016. We collected the NBS 17-OHP from both states, with 17-OHP levels obtained from NBS filter-paper dried whole-blood specimens (nM) multiplied by 66 to convert to serum concentration equivalents (ng/dL) [15]. Patients had a confirmed diagnosis of NCAH based on a baseline or stimulated 17-OHP level less than 303.03 nmol/L (10,000 ng/dL) [8] alongside clinical features at presentation; some were additionally genotyped.

In HOU, we also collected the NBS 17-OHP daily cutoff value at the time of screen, and the calculated difference between the NBS 17-OHP and cutoff value. The protocol for CAH NBSs in HOU includes a first screen collected between 24 and 48 h of life, and a second screen collected between 7 and 14 days of life. For the first NBS in newborns with a normal birthweight (≥2500 g), the top 5% of 17-OHP levels from that day’s newborn screens are retested, and the top 20% of those retested values are flagged as abnormal. For the second screen in newborns with a normal birthweight, the top 3% of 17-OHP levels from that day’s newborn screens are retested, and the top 15% of the retested values are flagged as abnormal. For newborns with a low birthweight (<2500 g), there is no separate screen or retest cutoff but rather a set cutoff 17-OHP level of ≥90 nmol/L on both the first and second screens.

In contrast to HOU, there is only one NBS performed by LA, similar to the majority of states in the U.S. The NBS is performed within the first 24–48 h of life and utilizes a two-tiered approach based on birth weight and 17-OHP on the initial screen (immunofluorescence assay). Results that meet the “urgent” cutoff criteria are labeled “abnormal”, whereas those that have indeterminate results (elevated 17-OHP but below “urgent” cutoff criteria) are retested by tandem mass spectrometry, along with androstenedione and cortisol, from the filter-paper card. Specimens with a high 17-OHP and an elevated ratio of 17-OHP + androstenedione divided by cortisol on the retest are considered abnormal.

The additional data collected on each patient included age at diagnosis, birth weight, gestational age, clinical presentation, relevant family history, ethnicity, genotype, and clinical data from follow-up visits.

### Statistical Analysis

The age at presentation and the 17-OHP levels are expressed as mean ± SD. The characteristics of patients who had a normal vs. an abnormal NBS in HOU and LA were compared using *t*-tests for continuous variables and Chi-Square or Fisher exact tests for categorical variables.

## 3. Results

Overall, 35 youth with NCAH in total from both centers were studied, 17 in HOU and 18 in LA, and their clinical presentations were evaluated. The age at diagnosis for youth with NCAH who were not identified by an NBS was 5.5 ± 3.1 years in HOU and 7.9 ± 4.0 years in LA (*p* = 0.16).

### 3.1. NCAH Patients in Texas

We studied 17 patients with NCAH in HOU (59% male; Table 1). There were no patients with a positive 17-OHP level on the first NBS (24.4 ± 9.0 nmol/L; state cutoff 58.2 ± 18.5; Table 2). Meanwhile, 52.9% percent (9/17) of patients were diagnosed with an abnormal second NBS at 16.4 ± 8.1 days old (17-OHP 59.4 ± 20.8 nmol/L; state cutoff 44.4 ± 16.2). The other half of the HOU cohort had lower (and negative) second NBS 17-OHP levels (36.0 ± 11.7 nmol/L; state cutoff 48.3 ± 12.0) compared with those with a positive screen (*p* = 0.01). Those patients not diagnosed by the NBS presented later in childhood with premature pubarche in 62.5% (5/8) and hypospadias/bifid scrotum in 12.5% (1/8). There were 25% (2/8) who were asymptomatic and diagnosed incidentally following a known sibling’s diagnosis of NCAH.

The race and ethnicity breakdown for patients in HOU was as follows: Caucasian 59% (10/17), African American 6% (1/17), Hispanic 29% (5/17), Middle Eastern 6% (1/17). There were no patients of reported Ashkenazi Jewish descent.

The ACTH stimulation test results were compared between NCAH patients identified via the NBS and those identified later in childhood (Table 2). There were no group differences in baseline 17-OHP levels for those identified via the NBS (7.4 ± 4.1 nmol/L) and those identified later in childhood (8.4 ± 3.1 nmol/L; *p* > 0.2). Those identified via the NBS had a stimulated 17-OHP level (110.1 ± 37.4 nmol/L) that was higher than those identified later in childhood (72.2 ± 35.4 nmol/L; *p* = 0.07), although not statistically significant. As the Texas cutoff for flagging 17-OHP abnormalities changed daily; days on which the cutoff was high likely led to cases not being caught that would have been flagged under a lower cutoff—possibly explaining the significant difference in state cutoff levels found between NCAH patients diagnosed by the NBS vs. clinically.

Genetic testing in patients identified by the NBS showed that most (86% (6/7)) had one allele with a classical CAH variant ([16]; Table 1); five were heterozygotes for a variant typically associated with NCAH and a classical CAH variant; one patient had two classical CAH variants. One patient was homozygous for a V281L variant, which is known to be frequently associated with NCAH. We note that the genetic results include pathogenic variants that are associated with classical CAH. Patient 2 was categorized as NCAH due to a stimulated 17-OHP of 109.08 nmol/L or 3600 ng/dL and a lack of adrenarche or hyperandrogenism at 8 yrs of age. The genotype of patient 3 included the P453S pathogenic variant, which has been associated with NCAH [17].

Conversely, genetic testing in five patients who were identified later in childhood showed that only 40% (2/5) had an allele with a classical CAH variant (Table 1); one was heterozygous for V281L and P30L (a variant associated with either NCAH or SV CAH). Two patients were homozygous for V281L variants.

Amongst the patients with NCAH identified by a second NBS, there were no cases of prenatal virilization, although 66.7% (6/9) eventually required hydrocortisone (HC) treatment (average age: 3.2 ± 3.9 years). One patient had premature pubarche at 4.8 years old; two patients had bone-age advancement; and three patients had biochemical abnormalities on stimulation testing. The other three patients not on hydrocortisone treatment were lost to follow up and included two females (1.5 and 13 years old) and one male (6 years old).

### 3.2. NCAH Patients in California

We studied 18 patients with NCAH in LA (17% male; Table 3). There were no patients diagnosed via an abnormal NBS (performed in the first week of life). All patients presented later in childhood (7.9 ± 4.0 years old). Most patients (66.7% or 12/18) presented with premature adrenarche, including one patient who had secondary central precocious puberty and three female patients with clitoromegaly. The other presentations included dysmenorrhea/amenorrhea (*n* = 2) and accelerated growth (*n* = 2). Two patients were asymptomatic and were diagnosed incidentally following a diagnosis of NCAH in a sibling.

Genetic testing was available in half of the patients identified in LA (Table 3). Two patients were homozygous for V281L; two were heterozygous for V281L and a common classical CAH variant; two were heterozygous for a V281L variant and either V281L or deletion on the second allele (as limited by polymerase chain reaction multiplex analysis); three patients had a rare variant on one allele, along with a common classical CAH variant on the second allele.

All LA patients were started on glucocorticoid replacement therapy upon diagnosis with NCAH, based on their clinical presentations related to hyperandrogenism in almost all cases. The age of treatment onset in CA was significantly higher than the age of onset of those identified in HOU by the NBS who eventually required treatment (HOU: 3.2 ± 3.9 years; LA: 7.9 ± 4.0 years, *p* = 0.02).

## 4. Discussion

Our main study findings include a high rate of detection of NCAH via a second NBS performed between 7 and 14 days of life, with the early identification of 53% of NCAH cases in HOU. Conversely, in LA there were no patients identified by only one state NBS. In addition, those neonates diagnosed with NCAH by the NBS had significantly higher 17-OHP values than those identified later in childhood by clinical presentation in the same state, suggesting that a second NBS is useful for identifying more severely affected patients as measured by biochemical markers. We note that, of the patients identified by the second screen, only one progressed to adolescence without the need for exogenous hydrocortisone.

Inherent differences between states in the number of newborn screens performed may also lead to differences in the clinical phenotype at presentation. Almost all children diagnosed clinically with NCAH were asymptomatic at birth but then commonly presented with premature pubarche later in childhood [6,18]. In LA, there were multiple children who presented with more virilized phenotypes in childhood, including clitoromegaly and/or secondary central precocious puberty. At HOU, in contrast, infants diagnosed by the second NBS were able to be followed closely by a specialist and were started on HC treatment earlier in childhood (average age of treatment onset 3.2 years), with only one patient who developed premature pubarche at 4.8 years old and subsequently started on HC. Therefore, the earlier identification of neonates with NCAH in HOU could prevent the more adverse effects of hyperandrogenism from developing, possibly by early monitoring of otherwise asymptomatic patients. In addition, a second NBS allowed for the identification of males (53% of identified infants in HOU), whereas those identified clinically later in childhood were 30.7% male (8/26, including 3 patients identified incidentally on screening after a female sibling was diagnosed). The diagnosis of males by the NBS is important as most adult males with NCAH are relatively asymptomatic and are identified only after a female family member is diagnosed. Thus, a second newborn screen could importantly identify males with NCAH who are much harder to identify on clinical presentation.

In addition, molecular genetic testing could allow for the classification and further understanding of the clinical severity in patients with NCAH. We and others have found that infants with NCAH identified on the NBS are typically compound heterozygotes for a classical and non-classical CAH variant [2,19] as was seen in 71% of our patients diagnosed by the NBS. The most common variant in our study was V281L. While the phenotype of NCAH would be expected to be determined by the activity of the less affected allele, there can be variability between different individuals for a given genotype [2,20,21]. Expensive genetic testing may not be available for all patients, such as children in LA with government-based insurance, which limits the number of patients with genotype data overall. Alongside the gold standard of baseline and stimulated 17-OHP values to screen and diagnose cases, broader access to genetic testing may play an important role in helping to identify missed cases of SV and NCAH [21,22,23,24,25]. In our retrospective analysis, we were able to examine the clinical phenotype of patients over time, which provided additional information. In Los Angeles, most study patients without genetic information were females who presented clinically between 5 and 7 years, had not exhibited virilized external genitalia as newborns, and had negative initial newborn screens. These factors make it much less likely that the females were missed cases of SV CAH.

Our study was limited by the relatively small sample size of patients affected with NCAH, the sampling bias within a two-center patient population, and the retrospective nature of the data collection. A more in-depth, longitudinal examination of this patient population, including patients who are identified on newborn screening versus later in childhood with a clinical diagnosis, would allow for the further quantification of symptomatology and age at onset. Cost–benefit analyses are limited in the literature but would be helpful given that the addition of a second NBS requires careful consideration of cost of screening [26]; the goals of screening should be clear and balance the cost of a second screen with those related to future morbidity due to excess androgens. Additionally, though this was not evaluated in our study, the potentiality of false-positive screening and increased anxiety within families should be considered—all of these aforementioned issues could implicate the weaknesses of a two-stage testing procedure as described in the Wilson and Junger criteria for screening [27]. In support of a second screen, nevertheless, we have shown that patients with NCAH identified as neonates present with milder symptomatology than those identified later in childhood and that these patients were able to obtain earlier treatment for their condition in HOU. Individuals with NCAH can show significant signs or symptoms of the disease beginning in childhood [6,8]. There can be ameliorating effects of glucocorticoid treatment on the consequences of hyperandrogenism, with potential benefit from earlier detection and monitoring of the condition in patients.

## 5. Conclusions

We show here that a second NBS is associated with the earlier identification of NCAH in neonates when compared to a single NBS. The early detection of NCAH can initiate monitoring, which, alongside treatment from a young age if clinically indicated, can ultimately help prevent the adverse consequences of hyperandrogenism later in childhood. Additional studies are needed to evaluate the potential reduction in morbidity from androgen excess with the early identification of neonates with NCAH, the role of genotyping, and the cost associated with a second newborn screen.

## Figures and Tables

**Table 1:** Clinical characteristics of patients with NCAH in Houston.

Patient	Sex	Age at First Endocrine Evaluation	Clinical Presentation	Race/Ethnicity	Family History of CAH	Baseline 17-OHP (nmol/L) [ng/dL]	Stimulated 17-OHP (nmol/L) [ng/dL]	Allele 1	Allele 2
**1**	M	7 weeks	Second NBS	Middle Eastern	No	4.42 [146]	85.52 [2822]	V281L	V281L or deletion
**2**	F	8 weeks	Second NBS	Caucasian	No	5.78 [191]	109.08 [3599.6]	I236K, V237E, M239K, F306 + t, Q318X	30 kb deletion
**3**	M	4 weeks	Second NBS	Caucasian	No	4.61 [152]	138.32 [4564.6]	P453S	Intron 2G
**4**	M	7 weeks	Second NBS	Hispanic	No	6.12 [202]	128.92 [4254.4]	V281L	Intron 2G
**5**	F	7 weeks	Second NBS	Caucasian	No	6.02 [199]	66.93 [2209]	N/A	N/A
**6**	M	8 weeks	Second NBS	Hispanic	No	2.59 [85.5]	74.68 [2464]	N/A	N/A
**7**	M	4 weeks	Second NBS	Caucasian	No; mother has short stature, hirsutism, PCOS	14.30 [471.9]	93.44 [3084]	V281L	30 kb deletion
**8**	F	16 weeks	Second NBS	Hispanic	No	9.21 [304]	187.52 [6188.2]	V281L	Intron 2G
**9**	F	5 weeks	Second NBS	African American	No	13.33 [439.9]	106.09 [3501]	V281L	Intron 2G
**10**	M	8 years	Premature pubarche	Caucasian	No	8.83 [291]	103.33 [3409.9]	V281L	P30L
**11**	F	5 years	Premature pubarche	Caucasian	No	10.39 [342.9]	66.17 [2184]	N/A	N/A
**12**	M	16 weeks	Hypospadias, bifid scrotum	Hispanic	No	13.63 [449.8]	66.91 [2208]	V281L	I172N
**13**	F	8 years	Premature pubarche	Caucasian	Brother with NCAH (i.e., patient 16)	3.56 [117]	54.99 [1815]	V281L	30 kb deletion
**14**	M	2 years	Asymptomatic; identified after sibling diagnosed with NCAH	Caucasian	Younger sister with NCAH	9.37 [309]	52.88 [1745]	V281L	V281L
**15**	F	5 years	Premature pubarche	Caucasian	No	7.78 [257]	133.74 [4413.4]	N/A	N/A
**16**	M	7 years	Asymptomatic; identified after sibling diagnosed with NCAH	Caucasian	Sister with NCAH (i.e., patient 13)	5.33 [176]	N/A	V281L	30 kb deletion
**17**	M	9 years	Premature pubarche	Hispanic	Sister with NCAH	8.45 [279]	27.22 [898.3]	V281L	V281L

Abbreviations: CAH, congenital adrenal hyperplasia; NCAH, non-classical congenital adrenal hyperplasia; N/A, not available.

**Table 2:** Patients with NCAH identified on NBS vs. identified in childhood in Houston.

	Patients Identified on Abnormal NBS (*n* = 9)	Patients Identified in Childhood; Normal NBS (*n* = 8)	*p*-Value
17-OHP on 1st NBS (nmol/L) [ng/dL]	22.1 ± 8.9 [1460 ± 590]	27.1 ± 8.8 [1790 ± 580]	0.26
Average daily state cutoff 17-OHP on 1st NBS (nmol/L) [ng/dL]	47.8 ± 11.1 [3160 ± 733]	70.0 ± 20.2 [4620 ± 1330]	0.01
17-OHP on 2nd NBS (nmol/L) [ng/dL]	59.4 ± 20.8 [3920 ± 1370]	36.0 ± 11.7 [2380 ± 772]	0.01
Average daily state cutoff 17-OHP on 2nd NBS (nmol/L) [ng/dL]	44.4 ± 16.2 [2930 ± 1070]	48.3 ± 12.0 [3190 ± 792]	0.57
Baseline 17-OHP (nmol/L) [ng/dL]	7.4 ± 4.1 [244 ± 135]	8.4 ± 3.1 [277 ± 102]	0.56
Stimulated 17-OHP (nmol/L) [ng/dL]	110.1 ± 37.4 [3633 ± 1234]	72.2 ± 35.4 [2383 ± 1168] (n = 6)	0.07
Patients with one allele with a classical CAH variant present	6 (of 7 genotyped)	2 (of 5 genotyped)	0.64

Data presented as mean ± standard deviation.

**Table 3:** Clinical characteristics of patients with NCAH in Los Angeles.

Patient	Sex	Age at First Endocrine Evaluation (Years)	Clinical Presentation	Race/Ethnicity	Family History of CAH	Baseline 17-OHP (nmol/L) [ng/dL]	Stimulated 17-OHP (nmol/L) [ng/dL]	Allele 1	Allele 2
**1**	F	5	Premature pubarche	Hispanic	Half-brother with CAH (same father)	29.7 [981]	N/A	N/A	N/A
**2**	M	5.5	Asymptomatic; identified after sibling diagnosed with NCAH	Asian/Caucasian, Ashkenazi Jewish	Sister with NCAH (i.e., patient #16)	35.79 [1181]	N/A	V281L	V281L
**3**	F	5	Precocious puberty	Hispanic	No	10.0 [330]	272.7 [9000]	N/A	N/A
**4**	F	7.6	Premature pubarche	Caucasian	No; sister is a carrier	120.8 [3987]	N/A	N/A	N/A
**5**	F	7.2	Premature pubarche	Hispanic	No	13.9 [459]	74.27 [2451]	N/A	N/A
**6**	F	5.2	Premature pubarche	Caucasian	No	167.3 [5520]	294.8 [9727]	N/A	N/A
**7**	F	6	Clitoromegaly, premature pubarche	Ashkenazi Jewish	No	60.36 [1992]	62.97 [2078]	V281L	V281L
**8**	F	6.3	Clitoromegaly, premature pubarche, body odor	Caucasian	No	162.5 [5362]	N/A	V281L	30 kb deletion
**9**	F	3.8	Asymptomatic; identified after sibling diagnosed with NCAH	Caucasian	Sister with NCAH (i.e., patient #8)	17.9 [591]	120.4 [3973]	V281L	V281L or deletion
**10**	F	4.5	Premature pubarche, acne, accelerated growth	Caucasian	No; mother had infertility issues	142.1 [4689]	253.3 [8360]	V281L, Q318X, R356W, F306 + 1nt cluster	Rare variant—polymorphism in intron 2
**11**	F	6.1	Premature pubarche, body odor	Hispanic	No	22.3 [735]	93.1 [3071]	Frameshift variant, exon 7	N/A
**12**	F	6.7	Clitoromegaly	Caucasian	No	301.2 [9941]	N/A	Y97D	In2G
**13**	F	7.7	Premature pubarche, body odor	Hispanic	No	67.58 [2230]	206.5 [6813]	N/A	N/A
**14**	F	17	Irregular menses, obesity	Hispanic	No	27.4 [904]	268.9 [8874]	N/A	N/A
**15**	M	8.4	Premature pubarche, body odor	N/A	No	68.30 [2254]	151.9 [5014]	30 kb deletion	Rare variant (specifics unavailable)
**16**	F	17	Amenorrhea	Hispanic	No; mother has oligomenorrhea and dysmenorrhea; 21 year old sister has oligomenorrhea and ovarian cyst	21.9 [723]	178.7 [5898]	N/A	N/A
**17**	F	14	Signs of hyperandrogenism	Hispanic	No; mother has oligomenorrhea	176.5 [5826]	57.58 [1900]	N/A	N/A
**18**	M	8.4	Accelerated growth	Caucasian	No	37.60 [2462]	181.2 [5978]	V281L	V281L or deletion

Abbreviations: CAH, congenital adrenal hyperplasia; NCAH, non-classical congenital adrenal hyperplasia; N/A, not available.

## Data Availability

The data presented in this study are available on request from the corresponding author. The data are not publicly available due to patient privacy concerns.

## References

[1] Speiser P.W., Azziz R., Baskin L.S., Ghizzoni L., Hensle T.W., Merke D.P., Meyer-Bahlburg H.F.L., Miller W.L., Montori V.M., Oberfield S.E. (2010). Congenital Adrenal Hyperplasia Due to Steroid 21-Hydroxylase Deficiency: An Endocrine Society Clinical Practice Guideline. J. Clin. Endocrinol. Metab..

[2] Tusie-Luna M.T., Traktman P., White P.C. (1990). Determination of functional effects of mutations in the steroid 21-hydroxylase gene (CYP21) using recombinant vaccinia virus. J. Biol. Chem..

[3] Speiser P.W., Dupont B., Rubinstein P., Piazza A., Kastelan A., New M.I. (1985). High frequency of nonclassical steroid 21-hydroxylase deficiency. Am. J. Hum. Genet..

[4] Hannah-Shmouni F., Morissette R., Sinaii N., Elman M., Prezant T.R., Chen W., Pulver A., Merke D.P. (2017). Revisiting the prevalence of nonclassic congenital adrenal hyperplasia in US Ashkenazi Jews and Caucasians. Rev. Endocr. Metab. Disord..

[5] Speiser P.W. (2009). Nonclassic adrenal hyperplasia. Rev. Endocr. Metab. Disord..

[6] Nordenström A., Falhammar H. (2019). Management of Endocrine Disease: Diagnosis and management of the patient with non-classic CAH due to 21-hydroxylase deficiency. Eur. J. Endocrinol..

[7] Finkielstain G.P., Kim M.S., Sinaii N., Nishitani M., Van Ryzin C., Hill S.C., Reynolds J.C., Hanna R.M., Merke D.P. (2012). Clinical Characteristics of a Cohort of 244 Patients with Congenital Adrenal Hyperplasia. J. Clin. Endocrinol. Metab..

[8] New M. (2006). Nonclassical 21-Hydroxylase Deficiency. J. Clin. Endocrinol. Metab..

[9] Wasniewska M.G., Morabito L.A., Baronio F., Einaudi S., Salerno M., Bizzarri C., Russo G., Chiarito M., Grandone A., Guazzarotti L. (2020). Growth Trajectory and Adult Height in Children with Nonclassical Congenital Adrenal Hyperplasia. Horm. Res. Paediatr..

[10] Pang S., Murphey W., Levine L.S., Spence D.A., Leon A., Lafranchi S., Surve A.S., New M.I. (1982). A Pilot Newborn Screening for Congenital Adrenal Hyperplasia in Alaska. J. Clin. Endocrinol. Metab..

[11] Edelman S., Desai H., Pigg T., Yusuf C., Ojodu J. (2020). Landscape of Congenital Adrenal Hyperplasia Newborn Screening in the United States. Int. J. Neonatal Screen..

[12] Therrell B.L., Berenbaum S.A., Manter-Kapanke V., Simmank J., Korman K., Prentice L., Gonzalez J., Gunn S. (1998). Results of Screening 1.9 Million Texas Newborns for 21-Hydroxylase-Deficient Congenital Adrenal Hyperplasia. Pediatrics.

[13] Chan C.L., McFann K., Taylor L., Wright D., Zeitler P.S., Barker J.M. (2013). Congenital Adrenal Hyperplasia and the Second Newborn Screen. J. Pediatr..

[14] Held P.K., Shapira S.K., Hinton C.F., Jones E., Hannon W.H., Ojodu J. (2015). Congenital adrenal hyperplasia cases identified by newborn screening in one- and two-screen states. Mol. Genet. Metab..

[15] Mei J., Williams I. (2017). Centers for Disease Control and Prevention. Newborn Screening Quality Assurance Program, Second-tier Congenital Adrenal Hyperplasia Proficiency Testing Program (CAHPT) Quarterly Report. Volume 7, No.1. https://www.cdc.gov/labstandards/pdf/nsqap/nsqap_CAHFeb2017.pdf.

[16] Charmandari E., Eisenhofer G., Mehlinger S.L., Carlson A., Wesley R., Keil M.F., Chrousos G.P., New M.I., Merke D.P. (2002). Adrenomedullary Function May Predict Phenotype and Genotype in Classic 21-Hydroxylase Deficiency. J. Clin. Endocrinol. Metab..

[17] Helmberg A., Tusie-Luna M.T., Tabarelli M., Kofler R., White P.C. (1992). R339H and P453S: CYP21 mutations associated with nonclassic steroid 21-hydroxylase deficiency that are not apparent gene conversions. Mol. Endocrinol..

[18] Moran C., Azziz R., Carmina E., Dewailly D., Fruzzetti F., Ibañez L., Knochenhauer E.S., Marcondes J.A., Mendonca B.B., Pignatelli D. (2000). 21-Hydroxylase–deficient nonclassic adrenal hyperplasia is a progressive disorder: A multicenter study. Am. J. Obstet. Gynecol..

[19] Tajima T., Nakae K.F.J., Toyoura T., Shimozawa K., Kusuda S., Goji K., Nagashima T., Cutler G.B. (1997). Molecular Basis of Nonclassical Steroid 21-Hydroxylase Deficiency Detected by Neonatal Mass Screening in Japan. J. Clin. Endocrinol. Metab..

[20] Riedl S., Röhl F.-W., Bonfig W., Brämswig J., Richter-Unruh A., Fricke-Otto S., Bettendorf M., Riepe F., Kriegshäuser G., Schönau E. (2019). Genotype/phenotype correlations in 538 congenital adrenal hyperplasia patients from Germany and Austria: Discordances in milder genotypes and in screened versus prescreening patients. Endocr. Connect..

[21] Gidlof S., Wedell A., Guthenberg C., von Dobeln U., Nordenstrom A. (2014). Nationwide neonatal screening for congenital adrenal hyperplasia in sweden: A 26-year longitudinal prospective population-based study. JAMA Pediatr..

[22] Therrell B.L. (2001). Newborn screening for congenital adrenal hyperplasia. Endocrinol. Metab. Clin. North Am..

[23] Gidlöf S., Falhammar H., Thilén A., von Döbeln A., Ritzén M., Wedell A., Nordenström A. (2013). One hundred years of congenital adrenal hyperplasia in Sweden: A retrospective, population-based cohort study. Lancet Diabetes Endocrinol..

[24] White P.C. (2009). Neonatal screening for congenital adrenal hyperplasia. Nat. Rev. Endocrinol..

[25] Witchel S.F. (2019). Newborn screening for congenital adrenal hyperplasia: Beyond 17-hydroxyprogesterone concentrations. J. Pediatr..

[26] Brosnan A.C., Brosnan P., Therrell B.L., Slater C.H., Swint J.M., Annegers J.F., Riley W.J. (1998). A comparative cost analysis of newborn screening for classic congenital adrenal hyperplasia in Texas. Public Health Rep..

[27] Claahsen-van der Grinten H.L., Speiser P.W., Ahmed S.F., Arlt W., Auchus R.J., Falhammar H., White P.C. (2022). Congenital Adrenal Hyperplasia-Current Insights in Pathophysiology, Diagnostics, and Management. Endocr. Rev..

